# The prognostic role of C‐reactive protein in patients with head and neck squamous cell carcinoma: A meta‐analysis

**DOI:** 10.1002/cam4.3520

**Published:** 2020-11-17

**Authors:** Yanglan Chen, Rong Cong, Chengjian Ji, Wenhua Ruan

**Affiliations:** ^1^ Department of Stomatology The Children’s Hospital Zhejiang University School of Medicine National Clinical Research Center for Child Health Hangzhou Zhejiang Province China; ^2^ Department of Urology First Affiliated Hospital of Nanjing Medical University Nanjing China

**Keywords:** C‐reactive protein, head and neck squamous cell carcinoma, meta‐analysis, prognosis

## Abstract

**Background:**

The prognostic role of the C‐reactive protein (CRP) in head and neck squamous cell carcinoma (HNSCC) has not been well investigated. This meta‐analysis aimed to evaluate the prognostic relevance of elevated CRP levels in patients with HNSCC.

**Methods:**

A relevant literature search was performed in PubMed, Web of Science, and Embase up to September 1, 2020. The pooled odds ratio and hazard ratio (HR) with 95% confidence interval (CI) were applied to evaluate the difference in overall survival (OS), progress‐free survival (PFS), and cancer‐specific survival (CSS) between patients with high CRP and those without. The pooled odds ratio (OR) with 95% CI were used to assess the association between CRP and clinicopathological features.

**Results:**

A total of 17 studies, including 4449 patients, were included. Pooled results showed that an elevated CRP was associated with worse OS (HR = 1.48, 95% CI: 1.24‐1.77), CSS (HR = 1.85, 95% CI: 1.38‐2.46), and PFS (HR = 1.73, 95% CI: 1.38‐2.17). Male patients, lymph node metastases, and higher tumor stage were related to elevated CRP level (OR = 1.67, 95% CI: 1.34‐2.09; OR = 2.40, 95% CI: 1.44‐3.99; OR = 1.39, 95% CI: 1.12‐1.74).

**Conclusion:**

Our meta‐analysis demonstrated that an elevated pretreatment of CRP indicates poor prognosis in HNSCC. Therefore, CRP is an indicator of the prognosis of patients with HNSCC and can be recommended for assessing prognoses in clinical work.

## Graphical abstract

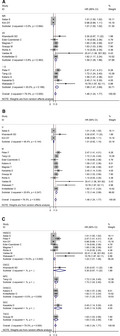

## 1 BACKGROUND

HNSCC is a frequent and global disease that includes the oral cavity, salivary gland, larynx, tongue, oropharynx, mucosal melanoma, maxillary sinuses, and nasopharynx. Over 600,000 new HNSCC cases are diagnosed worldwide annually, two‐thirds of which are already in a locally advanced stage. More than 300,000 patients are killed by HNSCC per year.^1^ Currently, worldwide, HNSCC is the fifth highest cause of cancer deaths among a variety of cancer types.^2^ In recent decades, HNSCC incidence has also increased due to the increasing prevalence of smoking.^3^

Given that the vast majority of patients have HNSCC in an advanced stage, multiple treatments are required, including surgery, RT, CT, and treatment with biological or targeted agents. Current standard treatments for locally advanced HNSCC involve surgery and postoperative adjustment CRT. However, postoperative CRT has potential adverse effects, including a high burden of toxicity to and morbidity of patients with HNSCC. In addition, it is costly for patients. Determining which cases will maximally benefit from adjuvant CRT after operation is vital. Therefore, new biomarkers that can be used to assess the prognosis of patients with HNSCC and guide clinical treatments must be discovered.^4^

Increasing evidence has proven that inflammatory response can promote the development and progression of carcinomas and affect the survival outcomes in patients with HNSCC. To date, some markers, including CRP, are demonstrated to be prognostic markers in various tumors. Over‐expressed CRP involves various tumors, including lung, lymphoma, ovary, and multiple myeloma.^5^, ^6^, ^7^, ^8^

CRP is a prototypic acute‐phase protein secreted by the liver into the blood. Usually, CRP is <2 mg/L in healthy individuals.^9^ In response to the acute inflammation or destruction of tissue cells, CRP plasma concentrations display a rapid and pronounced rise in the blood.^10^ The synthesis of CRP is stimulated by numerous factors, including bacteria, antigen‐immune responses, trauma, and fungi. CRP is also regulated by some pro‐inflammatory cytokines, such as interleukin‐6 (IL‐6), tumor necrosis factor, and interleukin‐1.^11^, ^12^ Meanwhile, CRP associated with systemic inflammatory processes has been shown to be a prognostic and predictive marker in various solid tumors, including renal, gastrointestinal, and hepatocellular carcinoma.^13^, ^14^, ^15^

In general, the ideal biomarker should be sensitive and specific; it must be relatively simple and noninvasive to detect tumor biomarkers in the blood of patients with cancer. Measuring CRP as a predictor in clinical practice is easy and cost‐effective. However, few studies have explored the role of CRP as a predictor of tumor recurrence and prognosis before the treatment of patients with HNSCC. Thus, this meta‐analysis aimed to evaluate the correlation between CRP and survival outcomes in patients with HNSCC.

## 2 MATERIALS AND METHODS

### 2.1 Search strategy

**FIGURE 1:**
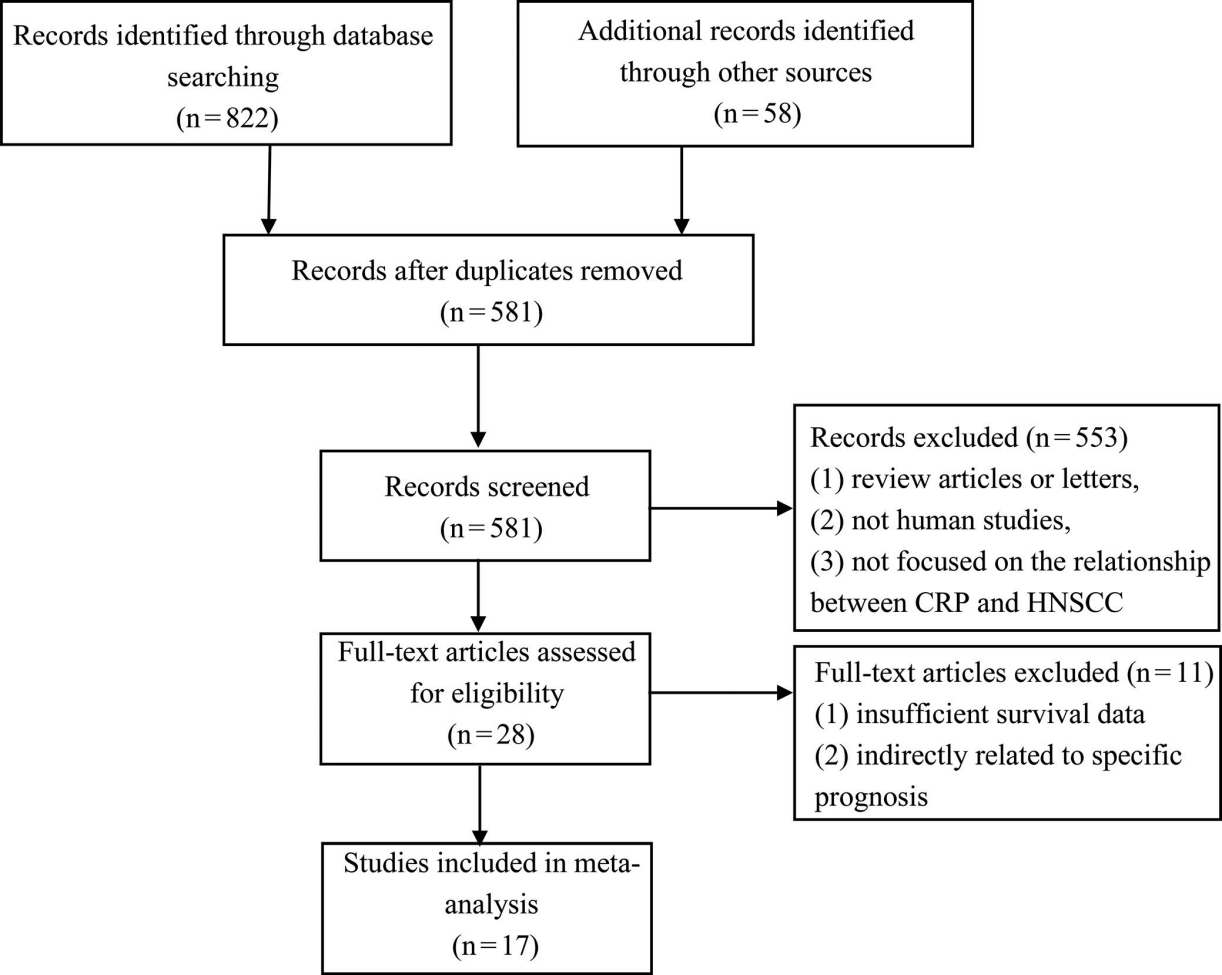
Flow diagram with details of the study selection process

PubMed, Web of Science, and Embase were searched for information on CRP and the outcomes of HNSCC up to September 1, 2020. Potentially relevant studies were searched using combinations of terms from the following sets of search terms: (“CRP” or “C‐reactive protein”), (“head” or “neck” or “head and neck” or “HNSCC” or “nasopharyngeal” or “laryngeal” or “gland” or “oropharyngeal” or “salivary duct” or “base of tongue” or “posterior pharyngeal wall” or “tonsil” or “soft palate”), and (“cancer” or “tumor” or “carcinoma”). In addition to these terms, the references within the retrieved articles were examined to identify additional studies relevant to the assessment in the present research. Figure 1 shows the flow diagram of the research selection for relevant studies in this work.

### 2.2 Inclusion criteria

Studies were included in this research if they met all of the following criteria. (a) The postoperative pathological diagnosis of each patient is HNSCC. (b) The CRP concentration in each patient's blood was evaluated before any treatment was administered. (c) The CRP pretreatment was measured using a serum‐based method. (d) The associations between high CRP expression in the patient's blood and clinicopathological analysis, prognosis, or HNSCC status were demonstrated in this study.

Studies were excluded from the present study based on the following criteria: (a) No relevant data could be extracted from the articles; (b) The study did not include survival outcomes or any useful HR with 95% confidence interval (CI); (c) Studies that contained no original experiments; (d) Studies that only reported elevated CRP or lowed CRP levels with no further explanation. In addition, to avoid pseudoreplication, when the data for the same patients appeared in more than one study and/or were combined with that of other patients, we only selected the most recent or the most complete study.

### 2.3 Data extraction and quality assessment

The relevant data were extracted from the eligible studies by two independent researchers (R.C) and (YL.C) and examined by a third researcher (CJ.J). The following relevant data were extracted: first author, year of publication, cancer type, country of patient population, gender, age, period of recurrence, median/mean age, follow‐up period, sample size of patients, tumor pathology, cancer stage, cut‐off value for CRP, and outcomes of patients, including overall survival (OS), cancer‐specific survival (CSS), progress‐free survival (PFS), and recurrence‐free survival (RFS). The Newcastle–Ottawa Scale was likewise applied to assess the quality of the included papers.

### 2.4 Statistical analysis

Statistical analysis was conducted using Stata software (12.0; Stata Corp LP). The relationships between CRP concentrations in the serum and the clinicopathological features, including gender of cases, tumor stage, and lymph node metastasis (LNM) of HNSCC cases, were evaluated by odds ratio (OR) along with 95% CI. For the prognostic effect of CRP, pooled HRs with 95% CI of CSS, RFS, OS, and PFS were used to evaluate the relationships between the CRP level survival outcomes of patients with HNSCC. We preferred to choose multivariate analyses when multivariate and univariate analyses of outcomes were available at the same time. If HR and 95% CI are not directly reported in the literature but Kaplan–Meier curves are provided, the HR with 95% CI can be directly obtained by extrapolating from the Kaplan–Meier curves. To evaluate the heterogeneity across the articles, we used the *χ* ^2^ and *I* ^2^ tests. *p* < 0.10 or *I* ^2^ > 50%, indicating significant heterogeneity, was considered optimal when using a random‐effects model, but not a fixed‐effects model, to estimate the pooled ORs/HRs. When *p* > 0.10 and *I* ^2^ < 50%, we preferred to use the fixed‐effects model. In addition, to detect the stability of the present results, a one‐way sensitivity analysis was performed by sequentially omitting each study included in this article. Begg test and funnel plots were adopted as well to evaluate any publication bias between the studies. All statistical tests were two‐tailed, and *p* < 0.05 (95% confidence limit) was considered statistically significant.

## 3 RESULTS

### 3.1 Search results and characteristics of included studies

On the basis of the criteria for inclusion in this research, 17 eligible studies, involving 4449 patients [1098 patients with HNSCC, 779 patients with oro‐hypopharynx squamous cell carcinoma (OHSCC), 306 patients with oral squamous cell carcinoma (OSCC), 197 patients with tongue squamous cell carcinoma (TSCC), 140 patients with salivary duct carcinoma (SDC), and 1668 patients with nasopharyngeal carcinoma (NPC)], were finally included in the meta‐analysis for evaluation.^16^, ^17^, ^18^, ^19^, ^20^, ^21^, ^22^, ^23^, ^24^, ^25^, ^26^, ^27^, ^28^, ^29^, ^30^, ^31^, ^32^ The detailed characteristics of the 17 eligible studies are shown in Tables 1 and 2. The patients with HNSCC originated from different countries, including Austria,^17^, ^20^, ^21^, ^30^ China,^27^, ^28^ France,^24^ Germany,^23^, ^31^ Japan,^19^, ^22^, ^29^ Korea,^16^ Switzerland,^25^ Taiwan,^18^ the United Kingdom,^26^ and Canada.^32^ The sample sizes of the included studies, which were retrospective and published between 2000 and 2020, ranged from 25 to 1589. The diverse treatments of patients with HNSCC included RT, CT, and CRT. The cut‐off value of CRP was 1.2‐10 mg/L in these studies. In the 17 articles, OS, PFS, CSS, and RFS were depicted in 14, 3, 3, and 3 studies, respectively.

**TABLE 1:** Main characteristics of studies included in this meta‐analysis

First author, publication year	Country	Recruitment period	Age (mean/median) yr	No.pts	Pathology	Stage	Survival analysis	Mean/median months of Follow‐up	NOS scores
Kim DY et al, 2016	Korea	2009‐2012	58	104	HNSCC	T1‐4N0‐3	RFS, OS	39	7
Eder‐Czembirek C et al, 2016	Austria	2004‐2014	58.04	144	HNSCC	T1‐4N0‐2	OS	84.1	8
DE Paz D et al, 2019	Taiwan	2010‐2016	53	246	OSCC	T1‐4N0‐3	NM	24	8
Katano A et al, 2017	Japan	2002‐2016	65	276	OHSCC	T1‐4N0‐3	CSS, OS	41	8
Graupp M et al, 2018	Austria	2002‐2015	58.9	197	TSCC	T1‐4N0‐3	DFS, OS	NM	7
Magnes T et al, 2017	Austria	2006‐2016	58	128	HNSCC	T1‐4N0‐3M0‐1	OS	NM	8
Kawakita D et al, 2017	Japan	1992‐2014	64	140	SDC	T1‐4N0‐3	PFS, OS	39.6	8
Peter F et al, 2012	Germany	2001‐2006	59	261	HNSCC	T1‐4N0‐3M0‐1	OS	48.2	6
Salas S et al, 2008	France	2003‐2006	59	72	HNSCC	T1‐4N0‐3M0	OS	21.8	6
Kruse AL et al, 2010	Switzerland	1999‐2008	NM	278	OSCC	NM	RFS	35.97	8
Khandavilli SD et al, 2009	UK	2002‐2005	NM	60	OSCC	T1‐4N0‐3	OS	NM	7
Tang LQ et al, 2015	China	2007‐2010	45	1589	NPC	T1‐4N0‐3M0	PFS, OS	44	6
Zeng YC et al, 2015	China.	2007‐2012	NM	62	NPC	T3‐4N0‐3M0	CSS	46.8	7
Rühle A et al, 2020	Germany	2010‐2018	72	246	HNSCC	T1‐4N0‐3M0‐1	OS	NM	8
Valdes M et al, 2020	Canada	2010‐2012	58	118	HNSCC	NM	RFS, OS	29.4	8
Wakasaki T et al, 2020	Japan	2017‐2019	65	25	HNSCC	NM	PFS, OS	10.1	8
Knittelfelder O et al, 2020	Austria	2000‐2017	58	503	OHSCC	T1‐4	CSS, OS	61	8

Abbreviations: CSS, cancer‐specific survival; HNSCC, head, and neck squamous cell carcinoma; NM, not mentioned; NPC, nasopharyngeal carcinoma; OHSCC, oro‐hypopharynx squamous cell carcinoma; OS, overall survival; OSCC, oral squamous cell carcinoma; PFS, progress‐free survival; RFS, recurrence‐free survival.

**TABLE 2:** HRs and 95% CIs of patient survival or cancer progression in association with CRP in eligible studies

First author, publication year	Cut‐off value (mg/L)	Case number		CSS/PFS/RFS HR (95%CI)	OS HR (95%CI)
		High expression	Low expression		
Kim DY et al, 2016	NM	NM	NM	0.98 (0.89‐1.11)	0.98 (0.89‐1.11)
Eder‐Czembirek C et al, 2016	10	40	104	NM	1.19 (0.6‐2.36)
DE Paz D et al, 2019	5	61	185	NM	NM
Katano A et al, 2017	3	144	132	1.989 (1.235‐3.203)	1.588 (1.069‐2.359)
Graupp M et al, 2018	5	84	66	1.454 (0.949‐2.227)	1.625 (1.032‐2.559)
Magnes T et al, 2017	8.5	NM	NM	NM	1.651 (1.058‐2.575)
Kawakita D et al, 2017	3.9	14	87	2.53 (1.28‐5.00)	2.45 (1.14‐5.3)
Peter F et al, 2012	2	113	148	NM	2.469 (1.414‐4.310)
Salas S et al, 2008	NM	NM	NM	NM	1.01 (1.00‐1.02)
Kruse AL et al, 2010	5	85	193	1.74 (1.08‐2.79)	NM
Khandavilli SD et al, 2009	5	42	18	NM	3.30 (0.97‐11.22)
Tang LQ et al, 2015	1.96	979	610	1.621 (1.273‐2.064)	1.723 (1.238‐2.398)
Zeng YC et al, 2015	8	18	44	3.04 (1.22‐7.55)	NM
Rühle A et al, 2020	5	NM	NM	NM	1.136 (0.548‐2.353)
Valdes M et al, 2020	6	NM	NM	4.974 (1.087‐ 22.755)	20.409 (1.380‐301.82)
Wakasaki T et al, 2020	1.2	11	14	2.932 (0.770‐11.148)	10.764 (1.869‐62.112)
Knittelfelder O et al, 2020	5	247	256	1.60 (1.08‐2.37)	1.62 (1.17‐2.24)

Abbreviations: CSS, cancer‐specific survival; HR, HR (high vs. low); NM, not mentioned; OS, overall survival; PFS, progress‐free survival; RFS, recurrence‐free survival.

### 3.2 Clinicopathological characteristics and CRP

**FIGURE 2:**
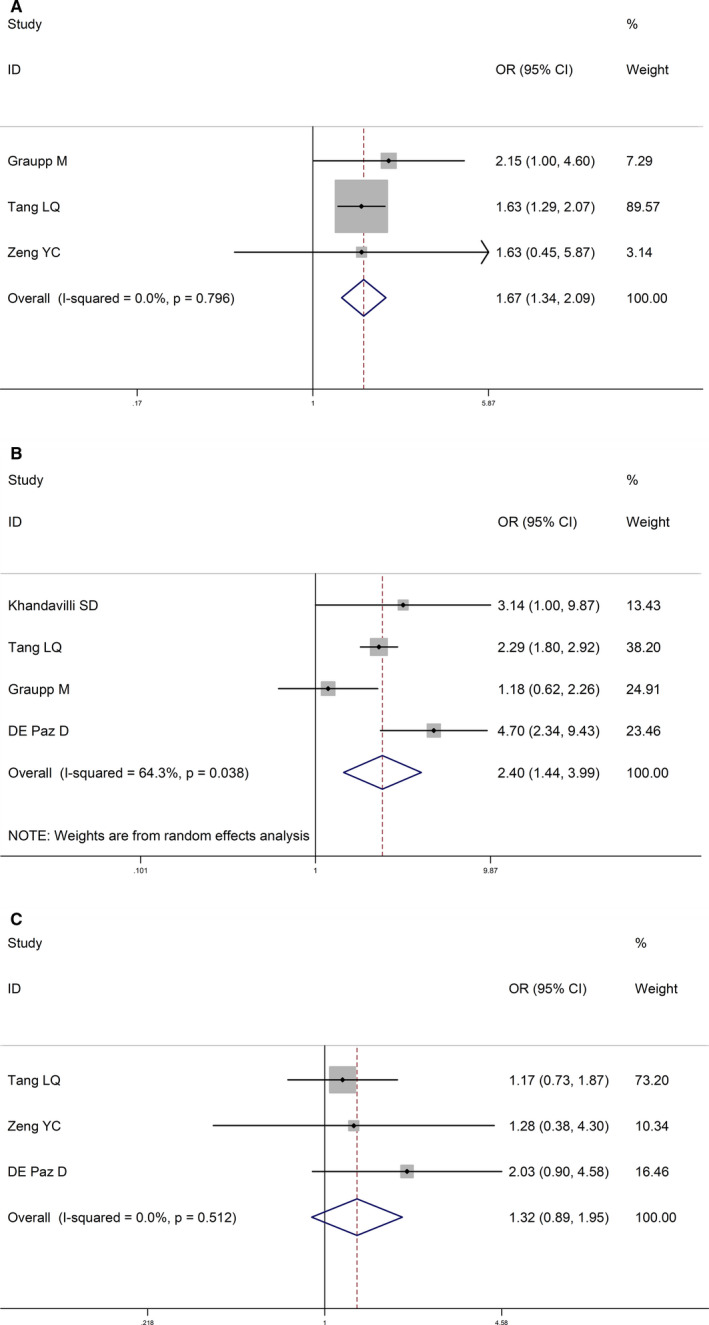
Forest plots for the association between CRP level and clinicopathological characteristics in HNSCC. (A) Association between CRP and gender in HNSCC. (B) Association between CRP and T stage in HNSCC. (C) Association between CRP and histology in HNSCC. HNSCC: head and neck squamous cell carcinoma; CRP: C‐reactive protein

The male patients and high tumor stage were associated with elevated CRP levels (OR = 1.67, 95% CI: 1.34‐2.09; OR = 2.40, 95% CI: 1.44‐3.99; Figure 2A,B; Table 3), demonstrated by this meta‐analysis with low heterogeneity was low (*I* ^2^ = 0.0%, *p* = 0.795; *I* ^2^ = 64.3%, *p* = 0.038; Figure 2A,B; Table 3). No significant difference was found between the CRP levels and the tumor grades (OR = 1.32, 95% CI: 0.89‐1.95; *I* ^2^ = 0.0%, *p* = 0.512; Figure 2C; Table 3).

**TABLE 3:** Association between CRP level and clinicopathological characteristics in HNSCC patients

Clinical parameters	Number of studies (number of patients)	OR (95%CI)	*p* value
Gender (male vs female)	3 (1865)	1.67 (1.34‐2.09)	<0.001
LNM (positive vs negative)	5 (2171)	1.39 (1.12‐1.74)	0.003
T stage (T1/2 vs T3/4)	4 (2092)	2.40 (1.44‐3.99)	0.001
Grade (G1/2 vs G3)	3 (1914)	1.32 (0.89‐1.95)	0.163

Abbreviations: CI, confidence interval; CRP, C‐reactive protein; OR, odds ratio.

**FIGURE 3:**
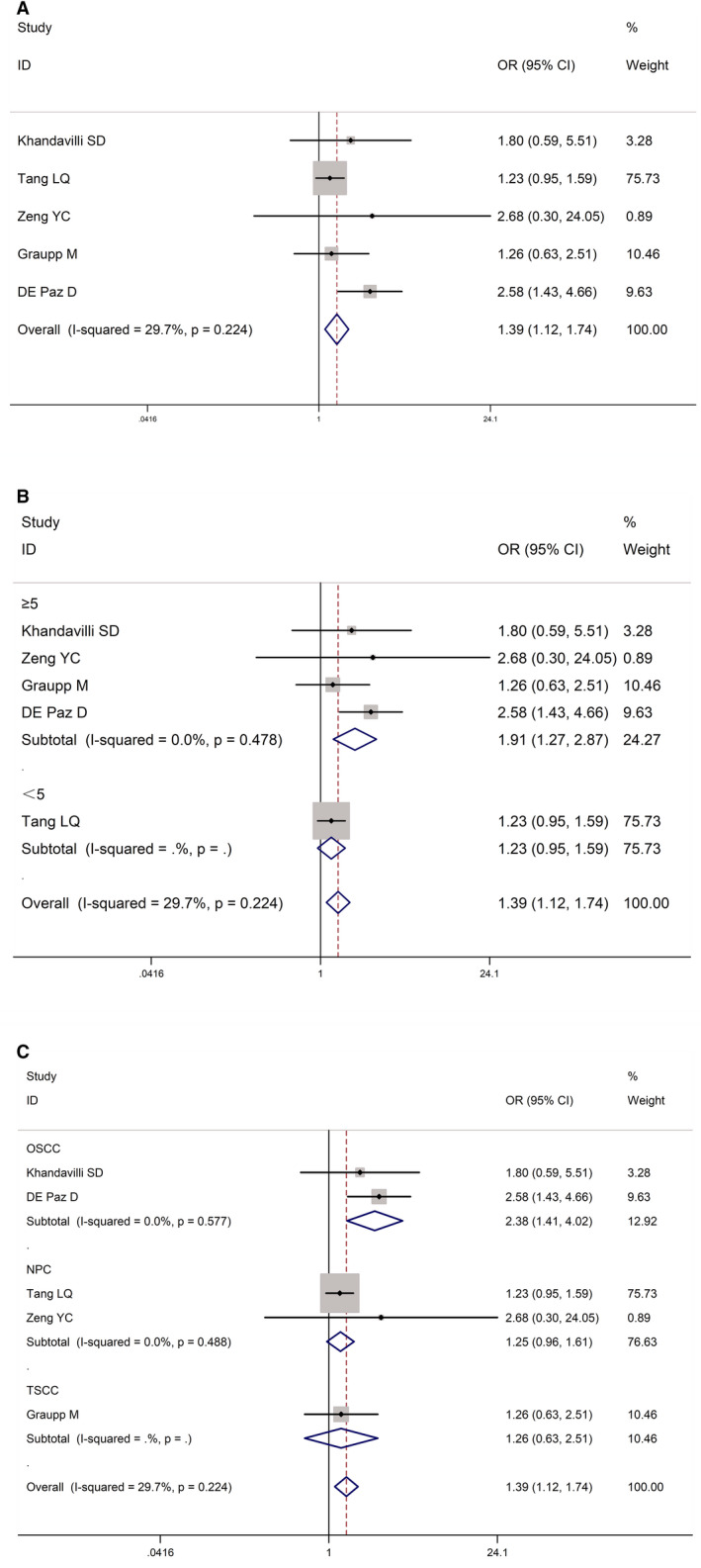
Forest plots for the association between CRP level and lymph node metastases in HNSCC. (A) Association between CRP and lymph node metastases in HNSCC. (B) Subgroup analysis of the association between CRP expression and lymph node metastases in HNSCC according to the cut‐off value of CRP. (C) Subgroup analysis of the association between CRP expression and lymph node metastases in HNSCC according to the tumor pathology. HNSCC: head and neck squamous cell carcinoma; CRP: C‐reactive protein.

The relationship between LNM and CRP was assessed in five studies, which involved 2171 patients. The pooled OR in this study indicated that cases with high levels of CRP were likely to develop LNM (OR = 1.39, 95% CI: 1.12‐1.74; *I* ^2^ = 29.7%, *p* = 0.224; Figure 3A). A subgroup analysis was performed on the basis of the cut‐off value of CRP owing to the high heterogeneity between the studies. When the cut‐off value of CRP was ≥5 mg/L, a significant difference was observed in the correlation between CRP and LNM (OR = 1.91, 95% CI: 1.27‐2.87; *I* ^2^ = 0.0%, *p* = 0.478; Figure 3B). A subgroup analysis was conducted according to the tumor pathology. In the group of OSCC cases, a significant difference was noted in the association between CRP and LNM (OR = 2.38, 95% CI: 1.41‐4.02; *I* ^2^ = 0.0%, *p* = 0.577; Figure 3C). However, no significant difference was observed between the CRP levels and LNM in the group of patients with nasopharyngeal carcinoma (OR = 1.25, 95% CI: 0.96‐1.61; *I* ^2^ = 0.0%, *p* = 0.488; Figure 3c).

### 3.3 Long‐term outcomes and CRP

**FIGURE 4:**
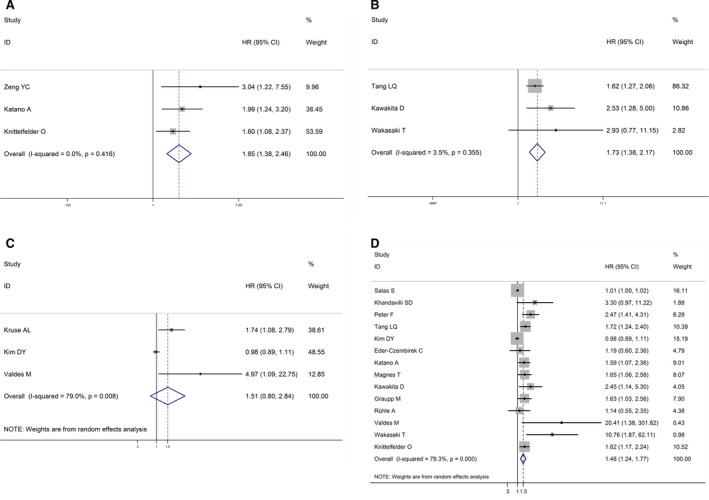
Forest plots for the association between CRP level and prognosis in HNSCC. (A) CSS. (B) PFS. (C) RFS. (D) OS calculated by multivariate analysis. CSS: cancer‐specific survival; RFS: recurrence‐free survival; HNSCC: head and neck squamous cell carcinoma; OS: overall survival; CRP: C‐reactive protein

Overall, high CRP levels were associated with worse CSS (HR = 1.85, 95% CI: 1.38‐2.46; *I* ^2^ = 0.0%, *p* = 0.416; Figure 4A) and PFS (HR = 1.73, 95% CI: 1.38‐2.17; *I* ^2^ = 3.5%, *p* = 0.355; Figure 4B) compared with patients with low CRP levels. It is shown no significant difference between high CRP levels and low RFS (HR = 1.51, 95% CI: 0.80‐2.84; *I* ^2^ = 79.0%, *p* = 0.008; Figure 4C). Meanwhile, using a random‐effects model after 14 studies were pooled, our meta‐analysis also suggested that the HNSCC cases with high CRP levels performed worse in OS compared with those who carried a low CRP (HR = 1.48, 95% CI: 1.24‐1.77, *I* ^2^ = 79.3%, *p* = 0.000; Figure 4D).

**FIGURE 5:**
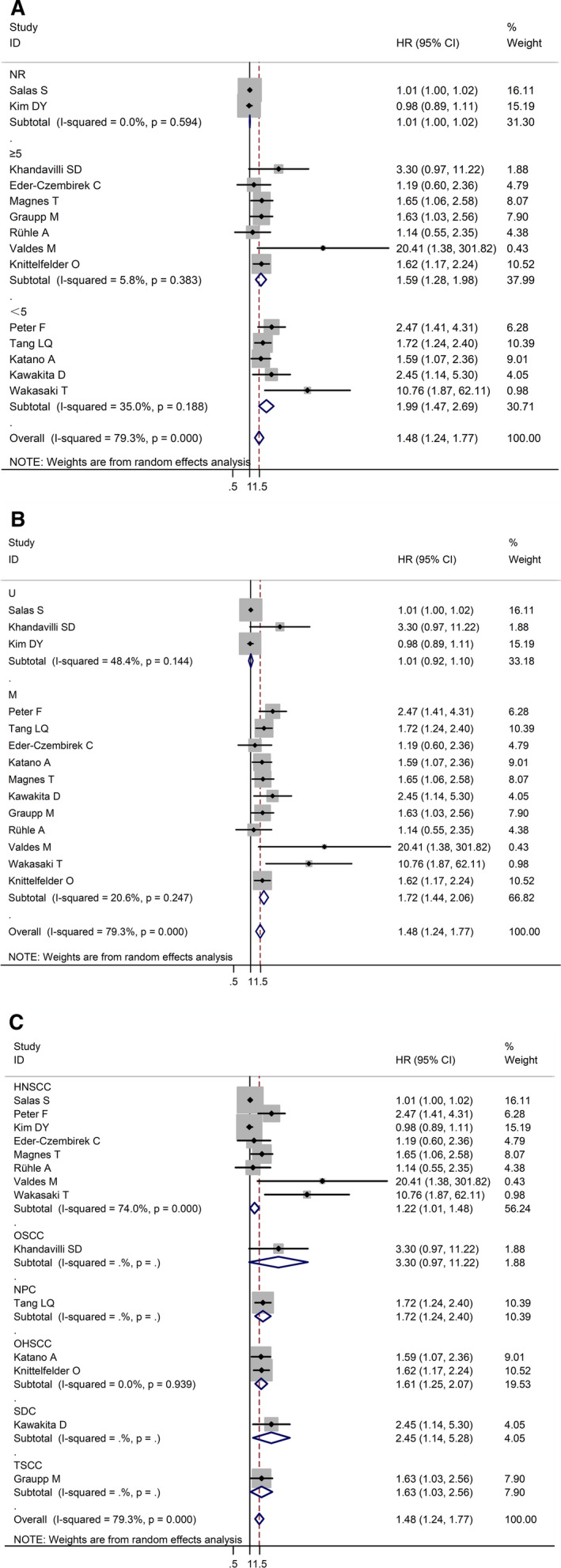
Subgroup analysis of the association between CRP expression and OS in HNSCC (A) according to the cut‐off value of CRP. (B) according to whether the value of HR was multivariate or univariate. (C) according to the tumor pathology. HNSCC: head and neck squamous cell carcinoma; CRP: C‐reactive protein

A subgroup analysis was performed on the basis of the cut‐off value of CRP. When the cut‐off value of CRP was ≥5 mg/L, a significant difference was observed in the correlation between CRP and OS (HR = 1.59, 95% CI: 1.28‐1.98, *I* ^2^ = 5.8%, *p* = 0.383; Figure 5A). When the cut‐off value of CRP was <5 mg/L, a significant difference was also noted in the correlation between CRP and OS (HR = 1.99, 95% CI: 1.47‐2.69, *I* ^2^ = 35.0%, *p* = 0.188; Figure 5A). Subgroup analysis was conducted according to whether the value of HR was multivariate or univariate. When HR took the univariate value, no significant difference was found in the correlation between CRP and OS (HR = 1.01, 95% CI: 0.92‐1.10, *I* ^2^ = 48.4%, *p* = 0.144; Figure 5B). When HR took the multivariate value, a significant difference was observed in the correlation between CRP and OS (HR = 1.72, 95% CI: 1.44‐2.06, *I* ^2^ = 20.6%, *p* = 0.247; Figure 5B). A subgroup analysis was conducted on the basis of the tumor pathology. In the group of HNSCC cases, which included eight studies, a significant difference was observed in the association between CRP and OS (HR = 1.22, 95% CI: 1.01‐1.48, *I* ^2^ = 74.0%, *p* = 0.000; Figure 5C).

### 3.4 Publication bias and sensitivity analysis

**FIGURE 6:**
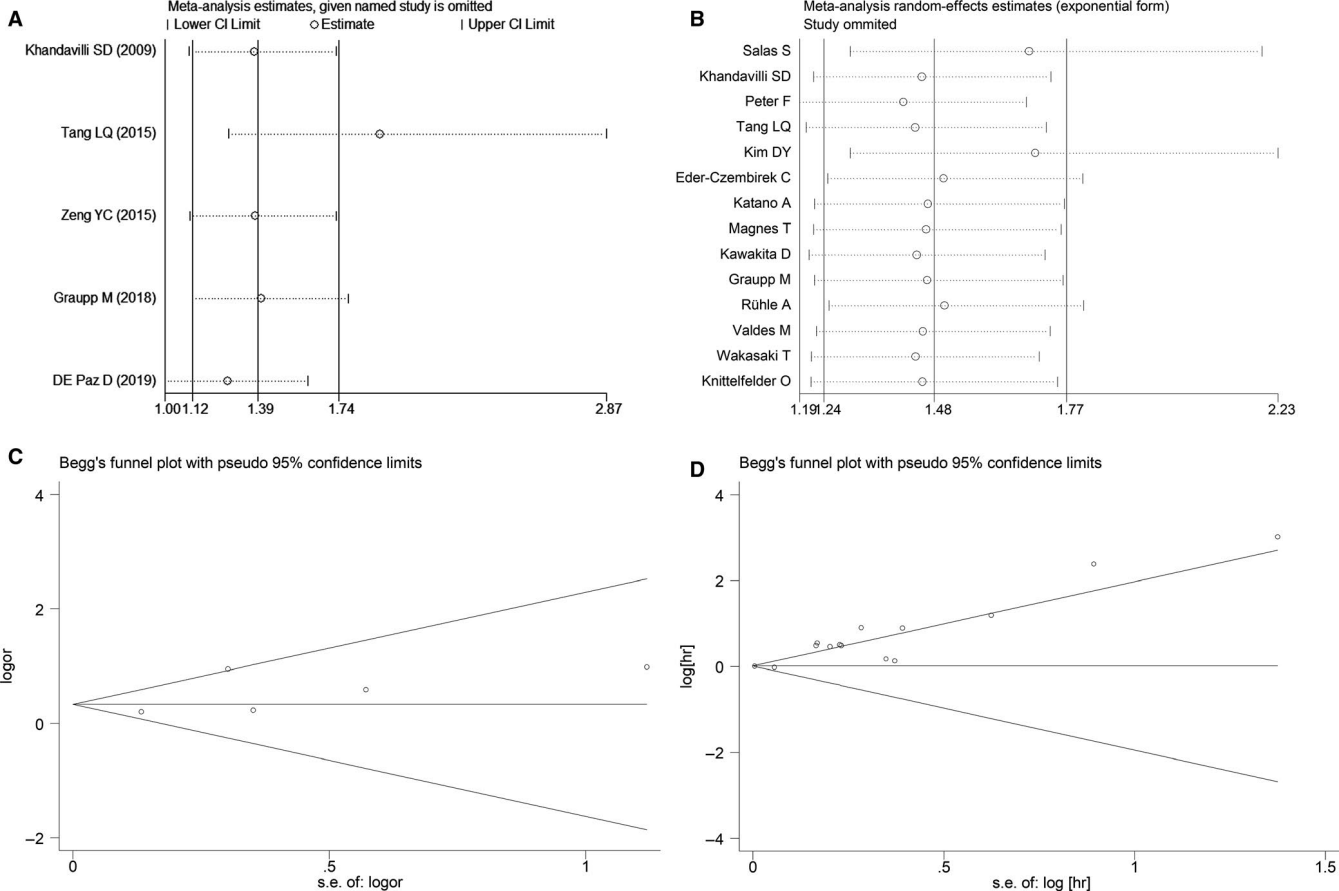
Publication bias and sensitivity analysis of each included study. (A) Sensitivity analysis for individual studies on the association between CRP and lymph node metastases; (B) Sensitivity analysis for individual studies on the association between CRP and OS; (C) Begg's plot for studies on CRP level and lymph node metastases; (D) Begg's plot for studies on CRP level and OS

A sensitivity analysis was applied to assess the contribution of each study to the estimations obtained from the pooled data. No obvious heterogeneity was observed, and sensitivity analysis again showed low heterogeneity in the included studies (Figure 6A,B). The funnel plot of Begg's test exhibited symmetricalness with a P value of 0.462 in LNM analysis (Figure 6C). Meanwhile, the funnel plot of Begg's test showed a P value of 0.743 in OS analysis (Figure 6D). no publication bias was found in our study, indicating the statistically reliable results of the meta‐analysis.

## 4 DISCUSSION

Worldwide, the majority of new cases of patients with HNSCC annually are related to smoking and alcohol consumption. The five‐year survival rate of patients with HNSCC is only approximately 50%, which is lower than those of other major carcinomas.^33^, ^34^ Sometimes, the behavior of the carcinoma and prognosis of the disease do not only depend on the clinical staging.^35^ An evaluation of the clinical and histological parameters, as published in many studies, revealed that some less advanced cases of HNSCC had an early recurrence, whereas other advanced cases had good survival rates.^36^, ^37^, ^38^ The TNM staging system is considered the most important prognostic factor for choice of therapy and prognosis, but it is still not accurate enough and should be supplemented with other indicators. Additionally, disease progression is associated with complex interactions between the tumor and the host's response to inflammation. The host's immune response releases a variety of mediators, which can promote chronic inflammation, cell proliferation, and even activation of unusual biochemical pathways. In tumor cases, these mediators eventually cause irreversible damage to the patient's DNA.^39^, ^40^ CRP is the first acute phase protein to increase, and within hours of the inflammatory response, it reaches concentrations thousands of times higher than normal.^41^ CRP has a short half‐life of only 6 h, and its concentration decreases rapidly when any tissue damage^42^ is not found. In the USA, CRP is the second and third most important cause of cancer‐related deaths among male and female patients, respectively.^43^

Increased IL‐6 is confirmed in patients with HNSCC. IL‐6 is secreted by certain cells, including t lymphocytes, tumor cells, and peripheral blood mononuclear and normal cells.^44^ Through the JAK–STAT signaling pathway, IL‐6 regulates the expression of genes associated with and proliferation.^45^ CRP is regulated by various cytokines like IL‐6.^41^ Elevated serum levels of IL‐6 have been proven to be associated with elevated serum CRP levels. IL‐6 can lead to inflammation and angiogenesis, which will subsequently raise the CRP levels.^44^ Jing Yang et al. found that CD32/FcgRII on myeloma cells can bind to CRP, subsequently activating the downstream signaling through the transcription factor and p38 mitogen‐activated protein kinase. This phenomenon results in increases in bone resorption and myeloma cell‐mediated osteoclast differentiation in vivo caused by osteolytic cytokines produced by myeloma cells.^46^ In Shasha Shen's study, CRP expression was upregulated in hepatocellular carcinoma (HCC) tissues compared with that in noncancerous tissues. The study demonstrated that the downregulation of CRP in HepG2 and Bel 7402 cells significantly inhibits cell growth, migration, and invasion in vitro, suggesting that high levels of CRP are carcinogenic in HCC. Moreover, it reported that the MEK/ERK and PI3K/AKT signaling pathways may be necessary for the CRP to stimulate cell migration and invasion of HCC cells.^47^ In 2013, Lin's study reported that cases with colorectal cancer (CRC) had a poor prognosis when CRP levels were above 5 mg/L. CRP can act as a LOX‐1 ligand in the CRC cell line. When the CRP concentration was 10 mg/L, the level of LOX‐1 positive cells on the cell surface increased by almost 13%, resulting in an elevation of important genes in CRC cases.^48^ These outcomes explained our results that elevated CRP levels are associated with LNM, high tumor stage, and worse prognosis, including CSS, PFS, and OS. In the subgroup analysis of CRP, when the cut‐off value of CRP was ≥5 mg/L, a significant difference was observed in the correlation between CRP, LNM, and OS. As mentioned above, increased CRP is a symbol of poor prognosis. No association between tumor grade and CRP levels was found. However, this result should be treated with caution as it may be based on a small sample size available for use. In the subgroup analysis of multivariate HR value, significant difference was observed in the correlation between CRP and OS.

Moreover, males obtain worse prognosis in some tumors, including CRC and gastric cancer. The immunological, genetic, environmental, and hormonal differences in gender contribute to this unique situation.^49^ Therefore, the high CRP in male patients with HNSCC may also be attributed to these gender differences. These outcomes explained our results that male patients are associated with elevated CRP levels, indicating a worse survival outcome.

There are some noteworthy limitations to our study. First, the type of cancer, staging, treatment strategy, and follow‐up month of patients with HNSCC varied, all of which may have an impact on the pooled results. Second, the cut‐off value of CRP varies from study to study, which might have led to heterogeneity. Third, our meta‐analysis included only 17 studies. Thus, high‐quality studies and a large sample size of studies are needed for verifying our results.

## 5 CONCLUSION

Our study revealed high pretreatment levels of CRP indicated poor prognosis for HNSCC patients. CRP could easily be measured by doctors before treatment and can assist in predicting the prognoses of patients with HNSCC and guiding their treatments. Combining CRP and tumor stage enhanced the predictive power of CRP. In other words, CRP can be a valuable prognostic biomarker for patients with HNSCC. However, to verify our suggestions, additional prospective studies are needed that include large sample sizes, different methods, and different populations. In addition, if our results are verified, the determination of CRP levels should become standard practice for assessing patients with advanced head and neck carcinoma.

## ETHICS APPROVAL AND CONSENT TO PARTICIPATE

Not applicable.

## CONFLICT OF INTERESTS

None.

## AUTHORS’ CONTRIBUTIONS

YL.C, R.C, and CJ.J performed the data extraction, analyzed the data, and wrote the manuscript. WH.R. helped to recheck the results and revised the manuscript. All authors read and approved the final manuscript.

## Data Availability

All the data used to support the findings of this study are included in the article. Please contact author for data requests.
